# The Cost of War Rations

**Published:** 1917-05-19

**Authors:** 


### FOOD IN WARTIME.*

### The Cost of War Rations.

Considerable ferment is being caused in institutions by the ever-increasing cost of food. Allowances which were considered with justice to err on the side of liberality in days of peace have now to be severely overhauled, and no two authorities seem to agree on the question of how much ought to be added to cover the universal rise in prices. The Lincoln Guardians, for instance, have, on the advice of the House Committee, sanctioned an increase from 10s. to 11s. 6d. per week in the cost of the officers' diet, the amount having already twelve months ago been raised from 9s. to 10s. At East Grinstead an increased grocery allowance of 4s. weekly per head has been made for each officer. On the other hand, the Grantham Guardians have reduced the officers' scale of rations from an approximate value of £1 per head per week to an approximate value of 16s., the actual cost of the food used being 14s. 10d. The Redruth Guardians having discovered that they are now saving 5s. 6d. per head per week on the officers' rations according to the war scale, have sanctioned a weekly grant of 4s. per head to each officer for extras. These examples illustrate the diverse ways in which Poor-Law institutions are managed. In cases where wasteful quantities of meat and other articles were formerly apportioned to the officers irrespective of whether or no they were actually required, the sharp curb imposed by war conditions has acted in the direction of economy, although the price of most commodities has increased from 30 to 50 per cent., and that of some necessaries has been doubled.

There is a strong desire in many quarters to have a standard rate of expenditure on food indicated, so that managers may know whether they are behaving with proper liberality to those under their administration, and at the same time are keeping within the limit of economy demanded by national difficulties. In old days we maintained and proved that for 1s. a day per head households of twenty and upwards could be provided with an ample and varied diet. What amount should be added to this estimate in these days of high prices? The British Red Cross Society has contributed useful information towards the solution of this pressing problem.

### Where Extravagance Commences.

Last year a Foodstuffs Advisory Board was formed in East Lancashire for the purpose of assisting in the catering at the seventy Red Cross and St. John Hospitals in this division of the county. The committee was composed of experts in foodstuffs and the allied trades, but does not appear to have numbered a woman caterer among its members. They issued a specimen menu for use in war hospitals, intended for soldiers in that stage of recovery when a generous diet is the first requirement, and their report (dated March 17, 1917) comments judiciously on such points as the time of meals, the serving of meals, prevention of waste, and the best manner of arranging the bill of fare for breakfast, dinner, tea, and supper. The menu provides meat or fish for breakfast, with porridge and bread and butter; for dinner, meat on six days in the week and fish on one, substantial puddings, and double (even treble) helpings of both meat and pudding; for tea, bread and butter and jam, with cake on Sunday, and occasionally such delicacies as brawn or toasted cheese; a light supper consisting of Army biscuits, milk, cocoa, bread and butter, cheese. Many hospital managers would get much instruction from perusing this bill of fare. They will possibly be surprised to learn that the experts lay it down as a fixed principle that at the present time the cost should not exceed twenty pence per man per day, or 11s. 8d. per week. This is no mere opinion passed on what things "ought to cost"; it has been worked out by men who had the opportunity of verifying their conclusions in the seventy hospitals of East Lancashire. They did not even assert that this was the minimum average cost of such fare as is indicated. They declared, on the contrary, that "anything over twenty pence is extravagant," and that hospitals of more than forty beds and over can be administered "more economically" than smaller ones.

### The Importance of Good Carving.

Managers of institutions will not go far amiss if they take this estimate of the cost of feeding wounded soldiers as a safe guide in rationing large numbers, whether of nurses or other officers. In respect to quantity there can hardly be two opinions about the fact that the average wounded convalescent requires a greater bulk of food than the ordinary man or woman doing civilian work. In respect of quality the experts in Lancashire tolerate nothing but the best. They reckon on intelligent economy in the use of all foodstuffs, but it is not the kind of economy which provides always just too little to satisfy hunger. Their advice about carving, for instance, if followed in all institutions, would raise the whole tone of the meals. "Money spent on good carving-knives repays itself quickly, and a good knife in the hands of a good carver will cover a big plate with 3½ oz. of meat, as against perhaps 6 oz. where an amateur carver is concerned. The appearance of a plate covered with meat has a high feeding value." The weekly bill of fare, which is too long to quote here, is admirably thought out, and admits of many variations. It would have been better to make it run ten days instead of seven, so that the days of the week were not labelled with a particular dish, for as they justly say, "'Monday, same old boiled mutton,' tends towards stagnation of recovery." It may safely be said, however, that were these meals copied exactly in any institution the catering would deservedly rank as first-rate.

Here, then, is the expert opinion as to the cost of catering for large numbers at the present time. The little delicacies which add much to the amenities of the nurses' or officers' table may be reckoned at 1s. 6d. per head per week, and count as a set-off to the difference in bulk of food required by convalescent soldiers. It follows, then, that a good housekeeper should be able to provide really excellent fare for the sum indicated by the East Lancashire Committee—namely, twenty pence a day or 11s. 8d. per week per head. We leave it to our readers to consider what monotonous, ill-cooked, badly planned, and slovenly served meals are frequently turned out at a cost exceeding by three to five shillings per week per head the above estimates.

* Previous articles appeared February 24, March 10, 24, and 31, April 14 and 21, May 5 and 12.

